# Supplementary material for: Effect of the Indentation Load on the Raman Spectra of the InP Crystal
Source: Materials (Basel). 2022 Jul 22;15(15):5098. doi: 10.3390/ma15155098 (PMC9331096; doi:10.3390/ma15155098)
Supplement: Supplementary file 1 [file materials-15-05098-s001.zip › materials-1815034-supplementary.pdf]

Supplementary Materials

Effect of the indentation load on the Raman spectra of the InP crystal

Dariusz Chrobak[1,*], Mateusz Dulski[1], Grzegorz Ziółkowski[2], Artur Chrobak[2]

[1] *Institute of Materials Engineering, Faculty of Science and Technology, University of Silesia in Katowice, 75 Pułku Piechoty 1A, 41-500 Chorzów, Poland*

[2] *Institute of Physics, Faculty of Science and Technology, University of Silesia in Katowice, 75 Pułku Piechoty 1A, 41-500 Chorzów, Poland*

* Corresponding Author, e-mail address: dariusz.chrobak@us.edu.pl

Supporting Figures

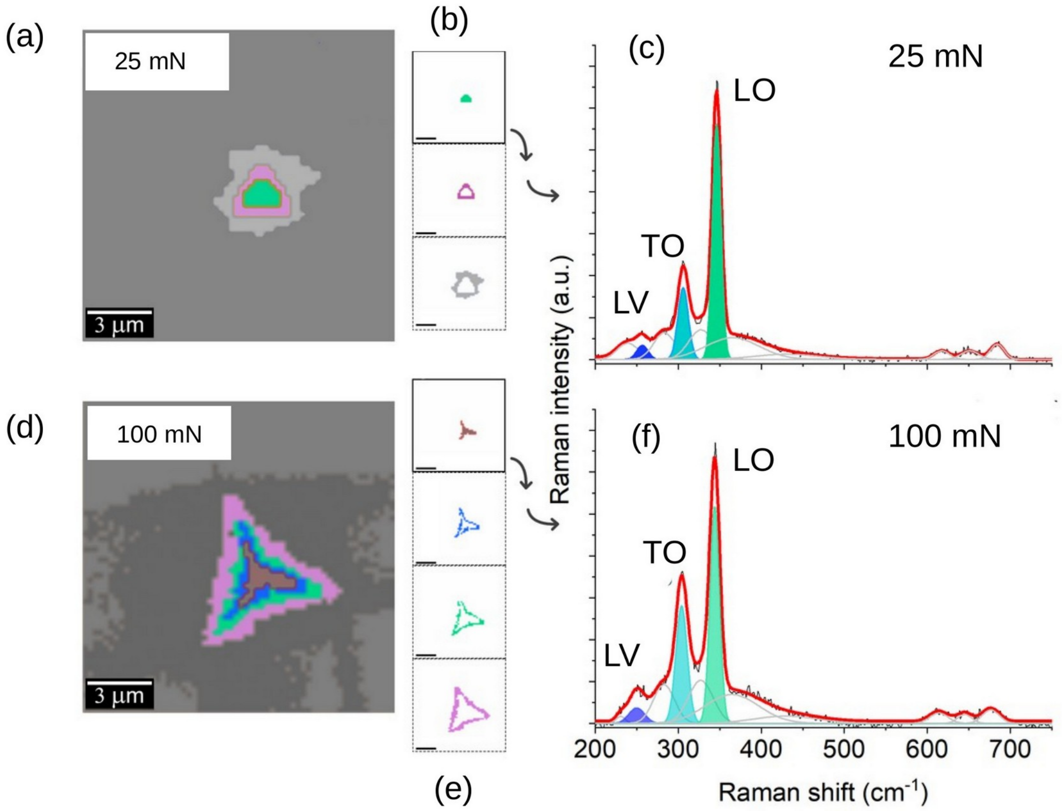

Figure S1 Exemplary K-means cluster analysis was performed on the base of Raman imaging around the S-doped InP crystal place indented at 25 mN and 100 mN loads. (**a**,**d**) An image of structural changes around the indents. (**b**,**e**) Individual clusters show the structural changes after indentation. (**c**,**f**) Raman spectra were obtained on the base of the signal exported from the most central fragment of the indent (arrows). Raman spectra were fitted using the Lorentz-Gauss function, while the color-highlighted peaks reveal the main LV, TO, and LO modes.

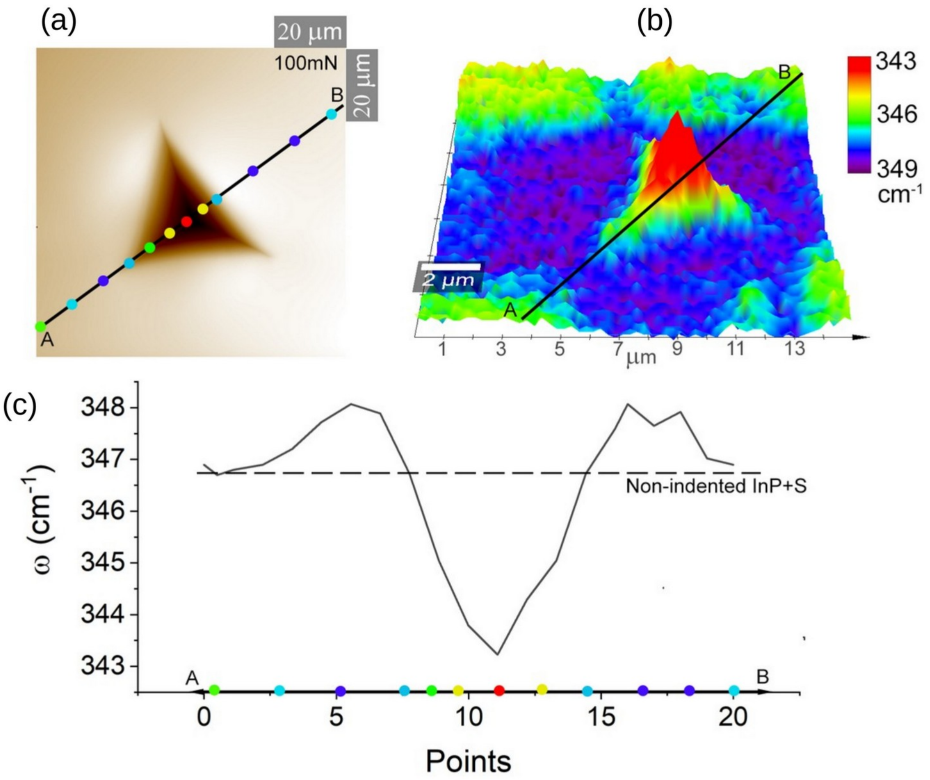

Figure S2 (**a**) The schematic image of the indent with cross-section and color highlighted points among the cross-section involving the information about the position of the LO mode. (**b**) Color-highlighted 3D Raman maps with a color corresponding to the position of the LO mode. (**c**) Cross-section profile through the indent prepared on the base of the position of LO mode. Data presented exemplary case of the S-doped InP indented with the maximum load 100mN.
